# Evaluation of antidiarrheal activity of ethanolic extract of *Holarrhena antidysenterica* seeds in rats

**DOI:** 10.14202/vetworld.2015.1392-1395

**Published:** 2015-12-11

**Authors:** Dushyant Kumar Sharma, Vinod Kumar Gupta, Surendra Kumar, Vivek Joshi, Ravi Shankar Kumar Mandal, A. G. Bhanu Prakash, Mamta Singh

**Affiliations:** Division of Medicine, Indian Veterinary Research Institute, Izatnagar, Bareilly, Uttar Pradesh - 243 122, India

**Keywords:** antidiarrheal, *Escherichia coli*, *Holarrhena antidysenterica*

## Abstract

**Aim:**

The present study was conducted to evaluate the antidiarrheal effect of ethanolic extract of *Holarrhena antidysenterica* (Family - Apocynaceae) seeds against induced diarrhea in Wistar albino rats.

**Materials and Methods:**

The extract was evaluated for castor oil and *Escherichia coli* induced diarrhea. Extract was given at 100, 200, 400 mg/kg body wt. orally in both protocols. Standard antidiarrheal Loperamide was used at 5 mg/kg body wt. orally in castor oil induced protocol, while standard antibiotic Gentamicin at 8 mg/kg body wt. intraperitoneally was used in *E. coli* induced diarrhea. In castor oil induced protocol, the percentage inhibition of defecation was calculated for each group, whereas in *E. coli* induced protocol, change in fecal consistency, and body weight was recorded for each individual rat for 3 days.

**Results:**

The severity of castor oil induced diarrhea was reduced significantly (p<0.05) with *H. antidysenterica* seeds extract at 200 and 400 mg/kg body wt. which showed equivalent effectiveness like that of Loperamide treated groups. Similarly in *E. coli* induced diarrhea protocol, the mean change in body weight was significantly (p<0.05) higher in positive control, whereas no significant variation was observed in negative control, Gentamicin treated and *H. antidysenterica* treated group at 200 mg/kg and 400 mg/kg body wt., respectively.

**Conclusion:**

The study concluded that ethanolic extract of *H. antidysenterica* seeds effectively controlled diarrhea and decreased the severity of clinical signs of castor oil and *E. coli* induced diarrhea in Wistar rats.

## Introduction

Diarrhea is the symptom of the gastrointestinal disorder, characterized by increase in stool frequency and alteration in consistency [1]. Diarrhea and the associated fecal urgency and incontinence result into imbalance between the absorptive and secretory mechanisms in the intestinal tract accompanied by hypermotility resulting in the excess loss of body fluids and electrolytes in feces [2]. Diarrhea is considered as the most imperative cause of death in neonatal farm animals worldwide. To nullify the problem of diarrhea in developing countries, the World Health Organization has constituted a diarrheal disease control program, which includes studies of traditional medicinal practices, together with health education and prevention strategies [3]. India is very rich harbor with reference to the diversity of higher plant species and also one of the leading countries in Asia with respect to a wealth of traditional knowledge system related to the use of plant species. Plants are very important sources of antidiarrheal drugs [4].

*Holarrhena antidysenterica* (Family- Apocynaceae, Common Name: Kutaza, Bitter Oleander, Kurchi) is a large tree, 9-10 meter long usually found in mountain areas. Seeds are linear or oblong concave with a long coma shape, light brown in color and show epigeal germination. Unani and Ayurvedic system of medicine utilizes this splendid herbal drug against many infectious diseases caused by helminthes, *Staphylococcus aureus*, *Entamoeba histolytica*, and *Escherichia coli* infections [5]. The bark and seeds of *Holarrhena* *antidysenterica* have been used for amoebic dysentery, diarrhea, asthma, bronchopneumonia, piles, eczema, fever, malaria, and diabetes mellitus [6,7]. *H. antidysenterica* produces gastrointestinal tract stimulation by activation of histamine receptors and relaxes the gastrointestinal tract by Ca^++^ channel blockade, which provided the basic ground for its usefulness in gut motility problems such as constipation, colic and diarrhea [8]. In past few years, due to the development of antibiotic resistance against infectious agents, plant kingdom has been extensively explored for its potential antimicrobial activity.

The aim of present study was to evaluate the antidiarrheal activity of *H*. *antidysenterica* seeds against infectious and non-infectious diarrhea in Wistar rats.

## Materials and Methods

### Ethical approval

The present study was given ethical clearance by Institutional Animal Ethical Committee. Animals were handled as per the guidelines of Committee for the Purpose of Control and Supervision of Experiments on Animals.

### Extract preparation

*H*. *antidysenterica* seeds were procured from local market. The seeds were authenticated by Dr. Dinesh K Saxena, Professor Emeritus (UGC), Department of Botany, Bareilly College, Bareilly. The voucher specimen (assigned no. 201401 02 2879 0332a) was deposited in college for further reference. The seeds were washed with distilled water, dried in shade, separated and made to dry powder. It was then passed through the 40 mesh sieve. Weighed amount of plant material was subjected to ethanolic columnar extraction in Soxhlet assembly as per standard protocol [9]. The extraction process was carried up to 12-15 cycles. The extract was dried at a temperature of 41°C and yielded brown colored powder weighing 8.3%, which was stored at 2-4°C for phytochemical analysis [10] and further use for evaluation of the antidiarrheal activity.

### Experimental animals, grouping and procedure

A total of 60 Wistar Albino rats (Indent voucher no. 6675) weighing 150-200 g were obtained from the Laboratory Animal Research Section (LAR) of IVRI, Izatnagar, Bareilly. The rats were kept in well ventilated experimental shed of medicine division under 12:12 light and dark hours in propylene cages. The animals were given weighed amount of standard feed supplied by feed section of IVRI and water *ad* *libitum*. Diarrhea was induced following two protocols with castor oil and *E. coli*.

### Castor oil induced diarrhea

The rats were divided into 6 groups of 5 rats in each group and acclimatized for 7 days in the experimental shed. Rats were fasted for 18 h before conducting the study. Group A-I received normal saline at 20 ml/kg body wt. served as negative control, Group A-II as positive control received normal saline at 20 ml/kg body wt., G A-III as standard treatment received Loperamide at 5 mg/kg body wt., and Group A-IV, Group A-V and Group A-VI were kept at 100 mg/kg, 200 mg/kg and 400 mg/kg body wt. *H. antidysenterica* seeds extract dosages for 3 days, respectively. All dosages were given orally. After 1 h of treatment, diarrhea was induced by oral gavage of castor oil (VASCO DRUG LABORATORIES, Agra) at 1 ml/rat in all groups except negative control [11]. All rats were transferred to propylene cages with absorbent paper and observed for watery wet or unformed stools for the next 4 h. A total number of wet or unformed droppings were counted and mean number of droppings/rat was calculated. The percentage inhibition of defecation was calculated for each group as per the following formula.

Percentage inhibition of defecation/drooping = ([A-B]/A) × 100

where A=Mean number of defecations caused by castor oil.

B=Mean number of defecation caused by drug or extract.

### E. coli induced diarrhea

The rats were divided into 6 groups of 5 rats in each group and acclimatized for 7 days in the experimental shed. Rats were fasted for 18 h before conducting the study. Group B-I and Group B-II served as negative control and positive control, respectively, and both received normal saline at 5 ml/kg body wt. Group B-III as standard treatment received antibiotic Gentamicin at 8 mg/kg body wt., and Group B-IV, Group B-V and Group B-VI, were kept at 100 mg/kg, 200 mg/kg and 400 mg/kg body wt. *H*. *antidysenterica* seeds extracts dosages respectively for 3 days. All dosages were given orally. The relevant strain of enterotoxigenic *E. coli* was obtained from the division of veterinary public health, IVRI, Izatnagar and grown on EMB agar. The *E. coli* suspension was made in physiological normal saline containing 2×10^8^ CFU/ml, which was calculated by spectrophotometric method. Diarrhea was induced in all groups except negative control by single oral administration of enterotoxigenic *E. coli* solution incubated at 37°C for 1 h at 5ml/kg body wt. The control group without induction of diarrhea received a single oral dose of physiological saline (vehicle) warmed at 37°C with an administration volume of 5 ml/kg [12]. Changes in fecal consistency and body weight were recorded for each individual rat.

### Statistical analysis

Data was subjected to statistical analysis using one-way ANOVA [13] and Tucky HSD SPSS software and p<0.05 was considered statistically significant.

## Results

The preliminary phytochemical analysis of *H*. *antidysenterica* seeds extract revealed the presence of alkaloid, carbohydrates, flavonoids, and phenolic compounds. Diarrhea was clinically apparent in all rats of positive control group after 30 min of oral administration of castor oil for next 4 h. Significant (p<0.05) percentage inhibition of defecation was recorded over 4 h with *H*. *antidysenterica* seeds extract given at 200 mg/kg body wt. (52.27±3.28) and at 400 mg/kg body wt. (54.18±1.37) compared to positive control group (Group A-II), whereas similar effect was produced by standard drug, Loperamide given at 5 mg/kg (Table-1).

**Table-1:** Effect of ethanolic extract of seeds of *H. antidysenterica* on castor oil induced diarrhea in rats.

Group (n=5)	Treatment	Total no. of wet dropping/cage in 4 h	Average no of wet dropping/animal in 4 h	% Inhibition of defecation
Group A-I	Saline at 20 ml/kg body wt.	0.00±0.00^a^	0.00±0.00^a^	0.00±0.00^a^
Group A-II	Saline at 20 ml/kg body wt.+1 ml castor oil	35.00±0.58^d^	7.00±0.12^d^	0.00±0.00^a^
Group A-III	Loperamide at 5 mg/kg body wt.+1 ml castor oil	15.00±0.58^b^	3.00±0.12^b^	57.17±0.94^c^
Group A-IV	HAE at 100 mg/kg body wt.+1 ml castor oil	25.00±0.58^c^	5.00±0.12^c^	28.47±2.83^b^
Group A-V	HAE at 200 mg/kg body wt.+1 ml castor oil	16.67±0.88^b^	3.33±0.17^b^	52.27±3.28^c^
Group A-VI	HAE at 400 mg/kg body wt.+1 ml castor oil	16.33±0.33^b^	3.26±0.06^b^	54.18±1.37^c^

Values are expressed as mean±SEM (n=5), and bearing different superscript was considered significant (p<0.05) when compared with control groups. HAE=Ethanolic extract of *Holarrhena antidysenterica* seed, SEM=Standard error of the mean, *H. antidysenterica=Holarrhena antidysenterica*

In the second protocol of *E. coli* induced, diarrhea was apparent in all groups except negative control (Group B-I) after 3.5 h of oral administration of *E*. *coli* suspension. The fecal consistency was found to be loose, semisolid in all groups on day 0 and continued to be loose until day 2^nd^ in all groups but on day 3^rd^ consistency became normal in all groups, except positive control (Group B-II) and *H*. *antidysenterica* seeds extract at 100 mg/kg (Group B-IV). No significant difference was observed in body weight of all groups on day 0. On the 3^rd^ day, a significant difference (p<0.05) was observed between body weights of the different groups. The body weight of *H*. *antidysenterica* at 200 mg/kg body wt. (Group B-V) and 400 mg/kg body wt. (Group B-VI) treated groups differed significantly (p<0.05) when compared to other groups, except negative control group (Group B-I) and Gentamicin treated group (Group B-III). The mean body weight change was significantly higher in the positive control (Group B-II), whereas negative control group (Group B-I), Gentamicin treated group (Group B-III), *H*. *antidysenterica* at 200 mg/kg body wt. (Group B-V) and 400 mg/kg body wt. (Group B-VI) did not show significant difference among them. A little drop in the food and water intake was observed in all groups except healthy control group (Table-2).

**Table-2:** Effect of ethanolic extract of seeds of *H. antidysenterica* on *E. coli* induced diarrhea in rats.

Group (n=5)	Treatment	Feacal consistency on 0 day	Feacal consistency on 3 day	Body weight (g)		Mean body weight change (g)

				0 day	3 day	
Group B-I	Saline at 5 ml/kg body wt.	Normal	Normal	187.0±5.24^a^	189.60±4.96^d^	6.60±1.69^ab^
Group B-II	Saline at 5 ml/kg body wt.	Loose semisolid	Loose semisolid	187.6±2.11^a^	161.80±1.35^a^	23.00±2.25^c^
Group B-III	Gentamicin at 8 mg/kg body wt.	Loose semisolid	Normal	185.2±2.71^a^	175.80±1.28^bc^	8.80±1.85^ab^
Group B-IV	HAE at 100 mg/kg body wt.	Loose semisolid	Loose semisolid	188.6±3.14^a^	169.00±2.23^ab^	14.00±1.34^b^
Group B-V	HAE at 200 mg/kg body wt.	Loose semisolid	Normal	190.2±2.61^a^	189.80±2.88^d^	3.80±0.73^a^
Group B-VI	HAE at 400 mg/kg body wt.	Loose semisolid	Normal	188.2±3.32^a^	184.60±2.11^cd^	6.00±2.60^ab^

Values are expressed as mean±SEM (n=5), and bearing different superscript was considered significant (p<0.05) when compared with control groups. HAE=Ethanolic extract of *Holarrhena antidysenterica* seed, SEM=Standard error of the mean, *H. antidysenterica=Holarrhena antidysenterica, E. coli=Escherichia coli*

## Discussion

Diarrhea is defined as increase in the fluidity, volume and frequency of bowel movements, increased secretion and decreased absorption of fluid, and thus loss of water and electrolytes from the body, and it is a hallmark sign of intestinal diseases [14]. The present study revealed the effect of *H. antidysentrica* seeds extract in castor oil induced diarrhea and *E. coli* induced diarrhea in rats. Castor oil contains an active ingredient ricinoleic acid, which is a hydroxylated unsaturated fatty acid, produced by action of lipases on castor oil in the upper intestine. The ricinoleate in the small gut lumen is poorly absorbed and it alters mucosal permeability, peristalsis and electrolyte transport (Na^+^ and Cl^–^) leading to hypersecreation and diarrhea. Ricinoleic acid stimulates epithelial cells to produce nitric oxide and adenylyl cyclase which lead to the production of prostaglandins (E series) induced diarrhea [15,16]. Therefore, inhibition of biosynthesis of prostaglandin is considered to inhibit the ricinoleate induced diarrhea [17]. *H*. *antidysenterica* seeds extract exhibits significant antidiarrheal activity. Plant extracts containing alkaloids, flavonoids, saponins, steroids, and tannins have been reported to possess antidiarrheal activity. Seed extract of *H*. *antidysenterica* was found positive for alkaloid and flavonoids which may be responsible for its antidiarrheal activity, comparable to standard antidiarrheal drug Loperamide. Enterotoxigenic *E. coli* produces a secretory-absorptive imbalance with no or little structural or mucosal damage. *E. coli* produces enterotoxin which increases the cyclic adenosine monophosphate responsible for enormous efflux of water and electrolytes producing watery diarrhea [18]. The present study revealed the significant antidiarrheal activity of *H*. *antidysenterica* seeds extract in *E. coli* induced diarrhea comparable to Gentamicin. It was reviewed that *H*. *antidysenterica* seeds extract encompasses potential antimicrobial activity against *Staphylococcus, Salmonella*, and *E. coli* and produces inhibition zone of 7.05 mm, 5.50 mm and 3.95 mm, respectively, on agar plate which is mainly because of the alkaloids conessine present in plant materials [19]. Our study revealed that *H*. *antidysenterica* seeds extract may serve as an effective antidiarrheal for gastrointestinal disorders.

## Conclusion

The present study concludes that ethanolic extract of *H*. *antidysenterica* seeds is effective against noninfectious as well as infectious diarrhea as it possesses antimicrobial and antidiarrheal property. Hence, it can be validated further for its future use.

## Authors’ Contributions

The study is the major component of the special problem of first author DKS. VKG provided the guidelines during the work and corrected manuscript. VJ, RSKM, and BPAG assisted in collection of samples and maintenance of experimental rats including weighing and medication. SK and MS assisted in analysis of data. All the authors have read and approved the final manuscript.
